# 5-Hydroxymethylcytosine profiles in plasma cell-free DNA reflect molecular characteristics of diabetic kidney disease

**DOI:** 10.3389/fendo.2022.910907

**Published:** 2022-07-29

**Authors:** Jin-Lin Chu, Shu-Hong Bi, Yao He, Rui-Yao Ma, Xing-Yu Wan, Zi-Hao Wang, Lei Zhang, Meng-Zhu Zheng, Zhan-Qun Yang, Ling-Wei Du, Yiminiguli Maimaiti, Gulinazi Biekedawulaiti, Maimaitiyasen Duolikun, Hang-Yu Chen, Long Chen, Lin-Lin Li, Lu Tie, Jian Lin

**Affiliations:** ^1^ College of Pharmacy, Xinjiang Medical University Key Laboratory of Active Components of Xinjiang Natural Medicine and Drug Release Technology, Urumqi, China; ^2^ State Key Laboratory of Pathogenesis, Prevention and Treatment of High Incidence Diseases in Central Asia, Urumqi, China; ^3^ Department of Nephrology, Peking University Third Hospital, Beijing, China; ^4^ Department of Pharmacology, School of Basic Medical Sciences, Peking University and Beijing Key Laboratory of Tumor Systems Biology, Peking University, Beijing, China; ^5^ Department of Pharmacy, Peking University Third Hospital, Beijing, China; ^6^ Synthetic and Functional Biomolecules Center, Beijing National Laboratory for Molecular Sciences, Key Laboratory of Bioorganic Chemistry and Molecular Engineering of Ministry of Education, College of Chemistry and Molecular Engineering, Innovation Center for Genomics, Peking University, Beijing, China; ^7^ Beijing Institute of Pharmacology and Toxicology, Beijing, China; ^8^ School of Food Science and Engineering, Hainan University, Haikou, China

**Keywords:** diabetic kidney disease, Epigenetics, 5-hydroxymethylcytosine 5-, cell-free DNDNA, biomarker

## Abstract

**Background:**

Diabetic kidney disease (DKD), one of the main complications of diabetes mellitus (DM), has become a frequent cause of end-stage renal disease. A clinically convenient, non-invasive approach for monitoring the development of DKD would benefit the overall life quality of patients with DM and contribute to lower medical burdens through promoting preventive interventions.

**Methods:**

We utilized 5hmC-Seal to profile genome-wide 5-hydroxymethylcytosines in plasma cell-free DNA (cfDNA). Candidate genes were identified by intersecting the differentially hydroxymethylated genes and differentially expressed genes from the GSE30528 and GSE30529. Then, a protein interaction network was constructed for the candidate genes, and the hub genes were identified by the MCODE and cytoHubba algorithm. The correlation analysis between the hydroxymethylation level of the hub genes and estimated glomerular filtration rate (eGFR) was carried out. Finally, we demonstrated differences in expression levels of the protein was verified by constructing a mouse model of DKD. In addition, we constructed a network of interactions between drugs and hub genes using the Comparative Toxicogenomics Database.

**Results:**

This study found that there were significant differences in the overall distribution of 5hmC in plasma of patients with DKD, and an alteration of hydroxymethylation levels in genomic regions involved in inflammatory pathways which participate in the immune response. The final 5 hub genes, including (CTNNB1, MYD88, CD28, VCAM1, CD44) were confirmed. Further analysis indicated that this 5-gene signature showed a good capacity to distinguish between DKD and DM, and was found that protein levels were increased in renal tissue of DKD mice. Correlation analysis indicated that the hydroxymethylation level of 5 hub genes were nagatively correlated with eGFR. Toxicogenomics analysis showed that a variety of drugs for the treatment of DKD can reduce the expression levels of 4 hub genes (CD44, MYD88, VCAM1, CTNNB1).

**Conclusions:**

The 5hmC-Seal assay was successfully applied to the plasma cfDNA samples from a cohort of DM patients with or without DKD. Altered 5hmC signatures indicate that 5hmC-Seal has the potential to be a non-invasive epigenetic tool for monitoring the development of DKD and it provides new insight for the future molecularly targeted anti-inflammation therapeutic strategies of DKD.

## Introduction

According to the latest report from the Diabetes Atlas of the International Diabetes Federation, the number of diabetic patients is expected to increase to 643 million by 2030 and 784 million by 2045. In 2021, diabetes has caused 6.7 million deaths, which means that one patient died every 5 seconds. Diabetic kidney disease (DKD) is one of the main microvascular complications of diabetes mellitus (DM) that remains a leading cause of end-stage renal disease (1). Worth mentioning, 30-40% of patients with DM develop DKD (2). Clinical examinations of proteinuria and estimated glomerular filtration rate are used to detect DKD (3). However, many patients with type 1 diabetes, and most with type 2 diabetes, do not follow this classic course in modern clinical practice. For example, many diabetic patients with renal impairment do not manifest excessive urinary albumin loss (4, 5). Discovering a set of non-invasive surrogate markers that reflect the progression of DM to DKD is urgently needed.

Previous publications have shown that genes related to the development of DKD are not only regulated by classical signaling pathways but also regulated *via* epigenetic mechanisms, including through chromatin histone modification, DNA methylation, and hydroxymethylation (6, 7). 5-hydroxymethylcytosine (5hmC), the oxidative product of 5-methylcytosine (5mC) catalyzed by ten-eleven translocation (TET) enzymes, is a relatively stable intermediate of active DNA demethylation and is regarded as an important epigenetic feature (8). The cfDNA is released into the blood circulation mainly through cell rupture and active DNA release mechanisms, which can reflect not only changes in the location of lesions but also changes in the immune system (9, 10). So there’s a fair amount of cfDNA epigenetic information that’s different in certain physiological conditions or diseases than it is in healthy people. 5hmC in plasma cfDNA could serve as more valuable biomarkers for non-invasive diagnosis and prognosis of various human diseases, such as cancers and cardiovascular disease (7, 11–13).

Herein, we set up a genome-wide, plasma cfDNA 5hmC profiling-based method for the distinguishment between DM and DKD patients. Plasma cfDNA from 17 DKD patients was subjected to 5hmC-Seal sequencing, and differential 5hmC markers associated with DKD were identified. The selected genes were further evaluated as biomarkers or potential therapeutic targets for DKD patients. This work may serve as preliminary research for the diagnosis of DKD using plasma cfDNA 5hmC marks, which could provide new insight for the future molecularly targeted therapy of DKD.

## Methods

### Study participants

All samples were collected at the Peking University Third Hospital. Informed consent was obtained from all participants before the study. Diagnosis of DM is based on the Diabetes Medical Care Standards published in 2020 by the American Diabetes Association (ADA) (14). Diagnostic criteria of DKD was according to the KDIGO 2020 Clinical Practice Guideline and the Consensus for Prevention and Treatment of Diabetic Kidney Disease 2014 of Chinese Diabetes Society and mainly includes urinary albumin-to-creatinine ratio (UACR) ≥30 mg/g and/or estimated glomerular filtration rate (eGFR)<60 mL·min⁻^1^·(1.73m^2^)⁻^1^, and lasts for more than three months (15).

### Acquisition of microarray data

**Figure 1:**
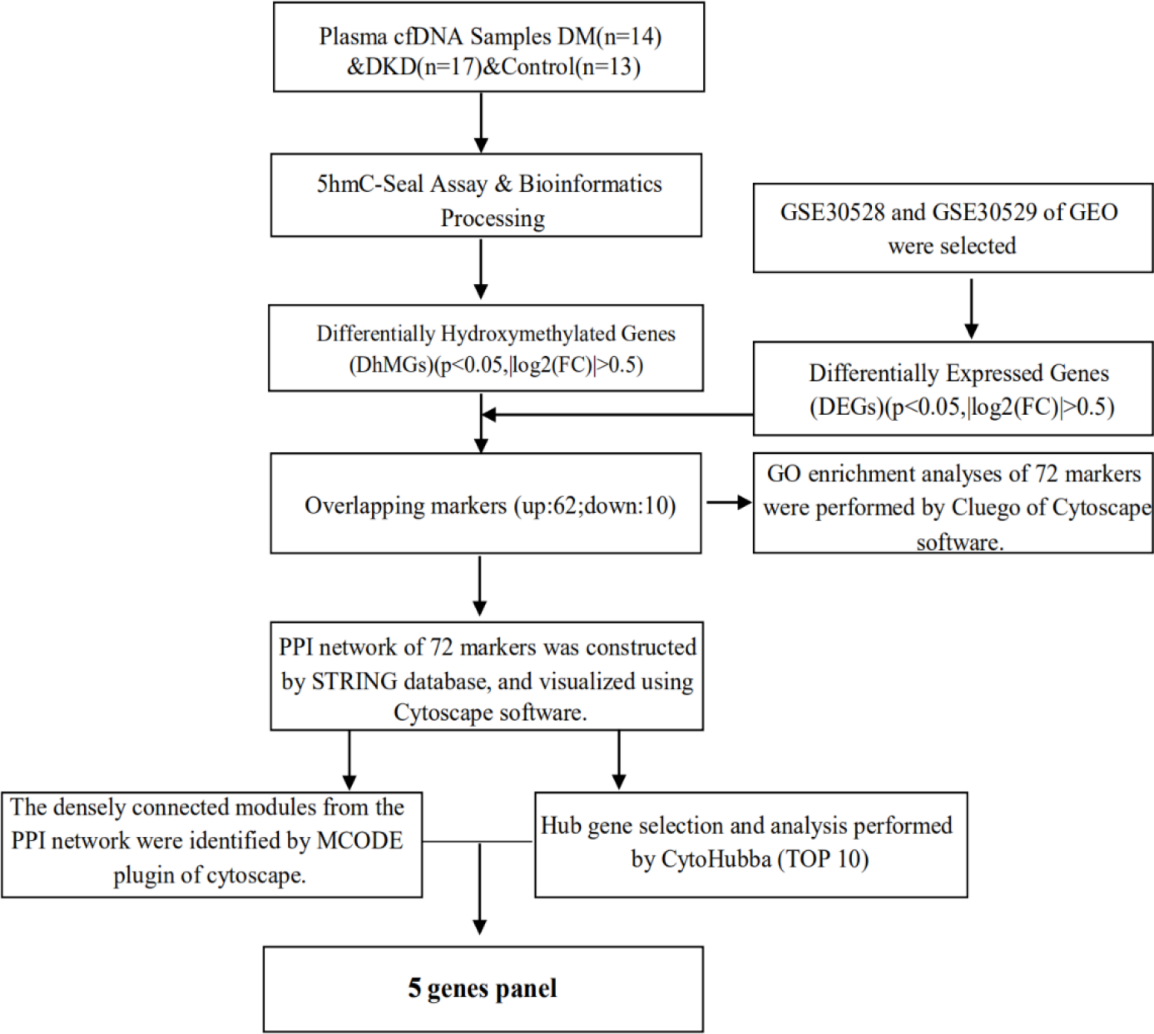
Overview of study design.

The mRNA expression and related experimental and clinical data of DKD were downloaded from GEO using the search terms diabetic kidney disease. The gene expression microarray datasets GSE30528 and GSE30529 were selected and downloaded. GSE30528 and GSE30529 were used for differentially expressed genes (DEGs) screening. Combined with the results of 5hmC-Seal, it was identified that the markers are also differentially expressed in DKD tissues. The detailed process of study design is shown in **Figure 1**.

### Clinical samples collection and cfDNA isolation

Whole blood specimens were obtained by routine venous phlebotomy and collected into Cell-Free DNA Collection Tubes (Roche). Tubes were maintained at 15°C to 25°C with plasma separation performed within 24h by centrifugation of whole blood at 1350×g for 15min at 4°C and 13,500×g for 5min at 4°C, followed by transfer of the plasma layer to a new tube. Plasma was aliquoted for subsequent cfDNA isolation or storage at −80°C. The plasma cfDNA was extracted using the QIAamp Circulating Nucleic Acid Kit (QIAGEN) following the manufacturer’s protocol and then stored at −20°C.

### 5hmC library construction and high throughput sequencing

5hmC library construction for all samples was consistent with previously reported (16, 17). The cfDNA samples were end-repaired using the KAPA Hyper Prep Kit (KAPA Biosystems) and then ligated to sequencing adapters. 5hmC bases were biotinylated *via* two-step chemistry and purified by the DNA Clean & Concentrator Kit (ZYMO), and subsequently enriched by binding to Streptavidin beads (Life Technologies). Then, the beads were resuspended in RNase-free water and amplified with PCR. Finally, the PCR products were purified using AMPure XP beads (Beckman). All libraries were quantified with a Qubit 3.0 fluorometer (Life Technologies). 5hmC sequencing was performed on the NextSeq 500 platform according to paired-end 39-bp high-throughput sequencing.

### Mapping and identifying 5hmC enriched regions

FastQC (version 0.11.5) was used to check the sequence quality. Raw 5hmC-Seal data were aligned to the human genome reference (hg19) with bowtie 2 (version 2.2.9) (18). Pair-end reads were extended and converted into BedGraph format and normalized the total number of aligned reads using Bedtools (version 2.19.1) (19), and then converted to bigwig format by using bedGraphToBigWig from the Integrated Genomics Viewer to visualize. Potential 5hmC enriched regions were identified using MACS2 (version 2.1.1) in each sample (20). Peak regions that appeared in more than 10 samples and that were less than 1000 bp were combined into one unified catalog for each patient. Genomic regions that tend to show artifact signals, according to ENCODE, were filtered out. The 5hmC enriched regions were generated by intersecting the individual peak call file with the merged peak file. We used the CHIP seeker package to annotate the 5hmC-enriched regions, and genes that were closest to the regions were used for the following analysis.

### Exploring functional relevance of DhMGs

After filtering genes in chromosome X and Y, differentially hydroxymethylated genes (DhMGs) in autosomes between DM and DKD samples were detected using the negative binomial generalized linear model in DESeq2 package (|log2FC| > 0.5 and *p*-value<0.05) (21). The enrichment analysis of the GO biological process (BP) was completed by the ClueGO (version 2.5.8) and CluePedia (version 1.5.8) plug-in from Cytoscape software (version 3.7.1). Medium network specificity, Bonferroni adjusted *p*<0.01, and enriched gene number>5 were chosen as the criteria for significance. The pathway enrichment analyses of DhMGs were performed using the Cluster Profile package in Bioconductor, and *p*-value<0.05 was considered statistically significant (22).

### Protein-protein interaction construction and selection of hub genes

We used the STRING database (https://string-db.org/) to conduct PPI for DhMGs (23). We mapped the DhMGs onto the PPI network and set an interaction score>0.4. Then, the Cytoscape software was used to visualize and construct the PPI network. To identify the key PPI network modules, the MCODE (version 1.6.1) plugin from the Cytoscape software was used to perform the gene network clustering analysis with the filter criteria of K-core=2, max depth=100, degree cut-off=2, and node score cut-off=0.2 (24). CytoHubba (version 0.1) was used to identify significant genes in the PPI network as hub genes (25). We used the maximal clique centrality (MCC), maximum neighborhood component (MNC), density of maximum neighborhood component (DMNC) algorithm to calculate the top 10 genes (26). Finally, all the results were intersected to obtain the final hub genes.

### Analysis of hub genes

Hub genes were analyzed by principal component analysis (PCA) using the FactoMineR package. Hierarchical clustering and heatmap analysis of DM and DKD on 31 samples were done by the pheatmap package. Correlation analysis between the clinical parameters and hub genes level was performed using Pearson correlation.

### Expression analysis of hub genes in mice

The hub genes protein expression levels were finally validated in DKD model of mice. Select 8-week-old male C57BL/6 mice, weighing 20-22g. At room temperature, the animals were reared in separate cages, fed with standard feed blocks with a protein content of 21%, and had free access to water. After 2 weeks, 60mg/kg STZ was intraperitoneally injected for five consecutive days, and the mice in the control group were intraperitoneally injected with sodium citrate buffer. After 96 hours, the tail was cut to collect blood, and the blood glucose concentration was measured, and those with blood glucose above 16.7mmol/L were selected as diabetic mice. The model cycle was 8 weeks and 16 weeks, respectively. Western blot (WB) method was used to verify the predicted protein expression of five hub genes in the mouse model.

### Drug-gene interaction network analysis

A drug-gene interaction network was constructed using the Comparative Toxicogenomics Database (CTD), an online database that provides information on gene-drug interactions for disease therapy and their relationship to disease (27). Networks were visualized with Cytoscape software 3.7.1.

### Statistical analysis

For clinical data, continuous data are presented as mean ± standard deviation, and categorical data are presented as numbers and percentages. Three groups were compared using one-way analysis of variance and Kruskal Wallis H test parameters and non-parametric continuous variables, while unpaired t-test and Mann Whitney U test parameters and non-parametric continuous variables were used for two groups comparisons. Subsequent analysis was carried out using R version 4.1.0.

## Results

### Clinical characteristics of DKD patients

Plasma samples from 14 DM patients, 17 DKD patients, and 13 healthy donors were collected. Clinical data were collected from all samples, and detailed information is listed in **Table 1** There were no statistically significant differences in subject age, gender, low-density lipoprotein (LDL), high-density lipoprotein (HDL), total cholesterol (TC), and triglycerides (TG) among the three cohorts used for discovery. In addition, related indicators of renal function, such as serum urea, creatinine, serum cystatin, eGFR, have statistically significant differences between the DKD and DM cases.

**Table 1:** Characteristics of the study subjects.

Characteristics	Control (n=13)	DM(n=14)	DKD(n=17)	P-Value
Age Mean (SD))	43.4 (7.2)	47.9 (9.9)	51.1 (16.7)	(0.257)
Gender (Male/Female)	6/7	9/5	9/8	0.902
Glucose (mean (SD)mmol/L)	4.2 (1.1)	8.9 (1.6)	8.3 (1.8)	<0.001
TC (mean (SD)mmol/L)	4.4 (0.7)	4.4 (0.7)	4.4 (0.5)	0.753
TG (mean (SD)mmol/L)	1.3 (0.5)	1.6 (0.4)	1.7 (1.0)	0.190
LDL (mean (SD)mmol/L)	2.85 (0.4)	2.5 (0.7)	2.5 (0.5)	0.212
HDL (mean (SD)mmol/L)	1.2 (0.4)	1.3 (0.4)	1.3 (0.2)	0.469
HbA1C (mean (SD),%)	–	7.6 (1.5)	6.8 (1.1)	0.054
Creatnine (mean (SD), µmol/L)	–	71.3 (12.8)	112.9 (44.4)	<0.001
Serum Urea (mean (SD), µmol/L)	–	5.8 (1.2)	7.8 (2.8)	<0.05
Cysc (mean (SD), mg/L)	–	0.7 (0.1)	1.4 (0.6)	<0.001
eGFR (mean (SD), mL/min1.73m^2^ mg/L)	–	98.4 (6.8)	53.9 (25.0)	<0.001
UACR (mean (SD), g/g)	–	–	2.5 (1.6)	–
24-h urine protein (mean (SD), g/d)	–	–	1.7 (0.4)	–

Data are presented as mean ± SD (standard deviation)TC, total cholesterol; TG, triglycerides; LDL, lowdensity lipoprotein cholesterol; HDL, high density lipoprotein; HbA1c, glycated hemoglobin; CysC, cystatin-C; eGFR, estimated ‘glomerular filtration rate; UACR, urinary albumin to creatinine ratio; DM, diabetes mellitus; DKD, diabetic kidney disease.

### 5hmC distribution in plasma cfDNA of DKD patients

**Figure 2:**
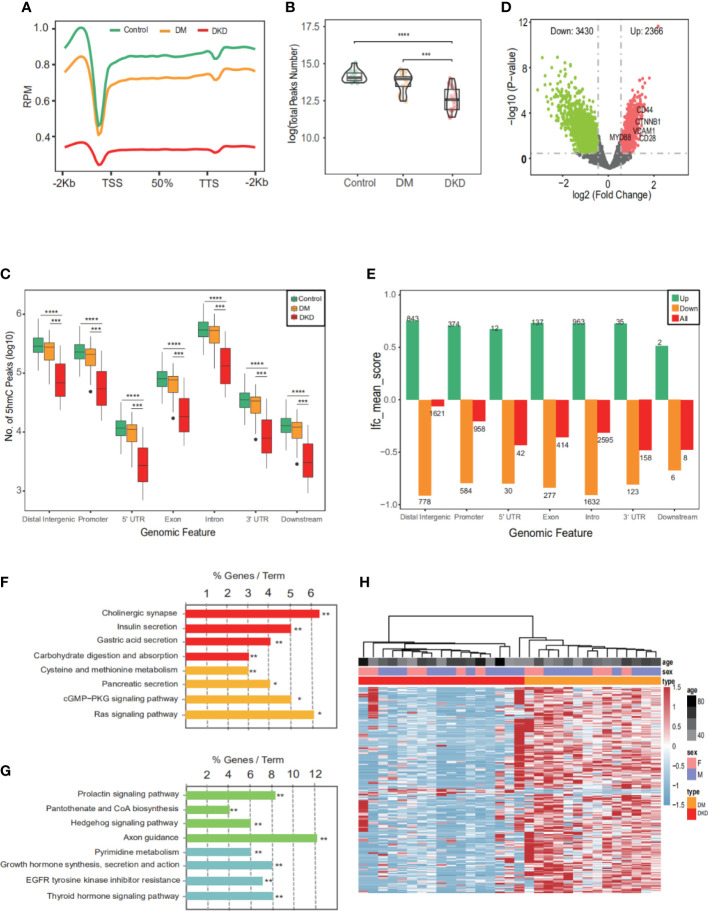
Characteristics of 5hmC distribution in plasma cfDNA of DKD patients. **(A)** The profiled 5hmC-Seal data in all samples cfDNA are enriched in gene bodies and depleted in the flanking regions. **(B)** Number of 5hmC peaks detected per million reads in Control, DM, and DKD cohorts. Each dot depicts an individual sample. **(C)** Genome-wide 5hmC distribution in different genomic features grouped by 3 groups (Control vs. DM vs. DKD). **(D)** Volcano plot. Significantly altered DhMGs (|log2FC| > 0.5, *p*-value <0.05) are highlighted in red (up) or green (down) using the DKD vs DM cfDNA samples. Grey dots represent the genes that are not differentially expressed. **(E)** Mean log2Foldchange value of 5796 DhMGs across different genomic features. **(F)** Pathways enriched in the upregulated marker genes with modified 5hmC between patients with and without DKD are shown. **(G)** Pathways enriched in the downregulated marker genes with modified 5hmC between patients. **(H)** Heatmap of top 200 DhMGs with sample type, age, and sex information labeled. Unsupervised hierarchical clustering was performed across genes and samples. RPM: Reads of exon model per Million mapped reads, *p<0.05, **p<0.01, ***p<0.001, ****p<0.001.

5hmC-Seal was performed with extracted cfDNA to map the genome-wide 5hmC profiles for all samples. First, we performed quality control (QC) analysis of 5hmC sequencing data from three sets of samples (Control, DM, DKD), such as mapping rate and number of reads (**Supplementary Figures S1A–D**). Then, sequencing data showed that 5hmC was enriched within transcription start sites (TSS) and transcription termination sites (TTS) and depleted in the flanking regions (**Figure 2A**), which was consistent with previous reports (11), suggesting that the accumulation of 5hmC is related to transcriptional activity. The total peaks detected per million reads in the DKD cfDNA cohort were significantly less than in DM and Control cohorts (**Figure 2B**). In addition, the overall 5hmC enrichment was significantly different between the three groups (Control, DM, DKD) for any genomic feature types (**Figure 2C**). Differential analysis between DM and DKD revealed 2366 hyper-hydroxymethylated genes and 3430 hypo-hydroxymethylated genes in DKD (**Figure 2D**, Additional file: **Supplementary Table 1**). The majority of these differential genes were enriched in intronic, intergenic, or promoter regions (**Figure 2E**), which was consistent with previous reports (28). The involving signaling pathways for the hyper-hydroxymethylated genes were classified in insulin secretion, carbohydrate digestion and absorption, and cysteine and methionine metabolism (**Figure 2F**). While the pathways for hypo-hydroxymethylated genes were included in growth hormone synthesis, EGFR tyrosine kinase inhibitor resistance, and Axon guidance (**Figure 2G**). A heatmap was generated by performing unsupervised clustering of the top 200 DhMGs between DM and DKD (**Figure 2H**). All findings indicate that these DhMGs may have the potential to distinguish patients with DKD from DM, and the overall 5hmC levels of these two groups of patients were significantly different.

### Function exploration related to immune response in the overlapping markers

**Figure 3:**
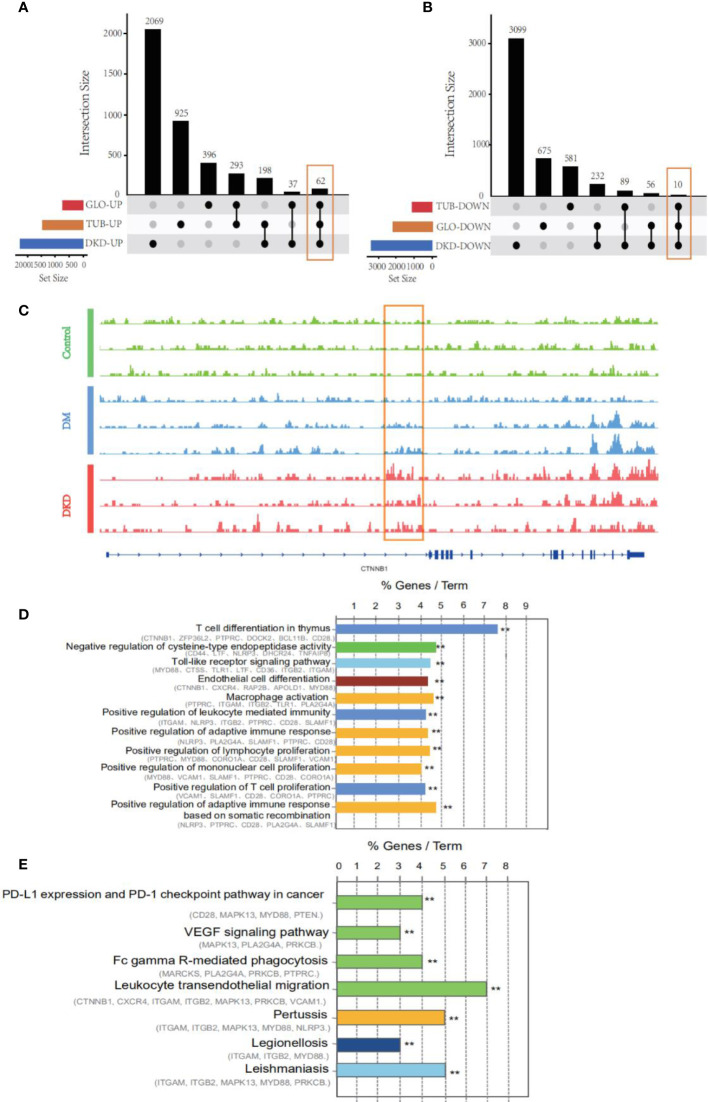
An alteration of hydroxymethylation levels in overlapping markers involved in inflammatory pathways which participate in the immune response. **(A)** An upset diagram of 62 intersected genes was found in upregulated genes *via* taking the intersection of DhMGs from 5hmC-Seal and DEGs from GSE30528 and GSE30529. DKD: 5hmC-Seal, TUB: GSE30529, GLO: GSE30528. **(B)** 10 intersected and downregulated genes among our cohort and GSE30528 and GSE30529. **(C)** IGV genome browser snapshot of CTNNB1 locus showing the increased 5hmC signal intensity in DKD samples compared to Control and DM. **(D)** GO enrichment analysis and function exploration of 72 DhMGs using Cytoscape software. (**p < 0.01). **(E)** KEGG pathways of 72 DhMGs using Cytoscape software. (**p < 0.01).

To examine the impact of DhMGs in the glomerular and tubular tissue of DKD patients, the 5hmC profiles were compared among DKD tubular (GSE30529, n=10) and glomerular (GSE30528, n=9) samples. A total of 72 overlapping gene markers were identified as kidney-related DhMGs (**Figures 3A, B**). One individual marker, catenin beta-1(CTNNB1), was randomly selected and tested with a set of randomly selected patients and healthy donors (**Figure 3C**), which proved the reliability of the identified gene panels. Pathway analysis of 72 overlapping markers in DKD patients suggested functional enrichment in certain canonical pathways. The top enriched GO included pathways involved in T cell differentiation in the thymus, negative regulation of cysteine-type endopeptidase activity, toll-like receptor, and endothelial cell differentiations (**Figure 3D**). Regarding KEGG pathways, the most enriched related pathways in the set of DhMGs were PD-L1 expression and PD-1 checkpoint pathway, VEGF signaling pathway, Fc gamma R-mediated phagocytosis, Leukocyte transendothelial migration (**Figure 3E**). These results show that the overlapping markers we screened are mainly related to the DKD immune response.

### Protein-protein interaction network construction and hub gene analysis

**Figure 4:**
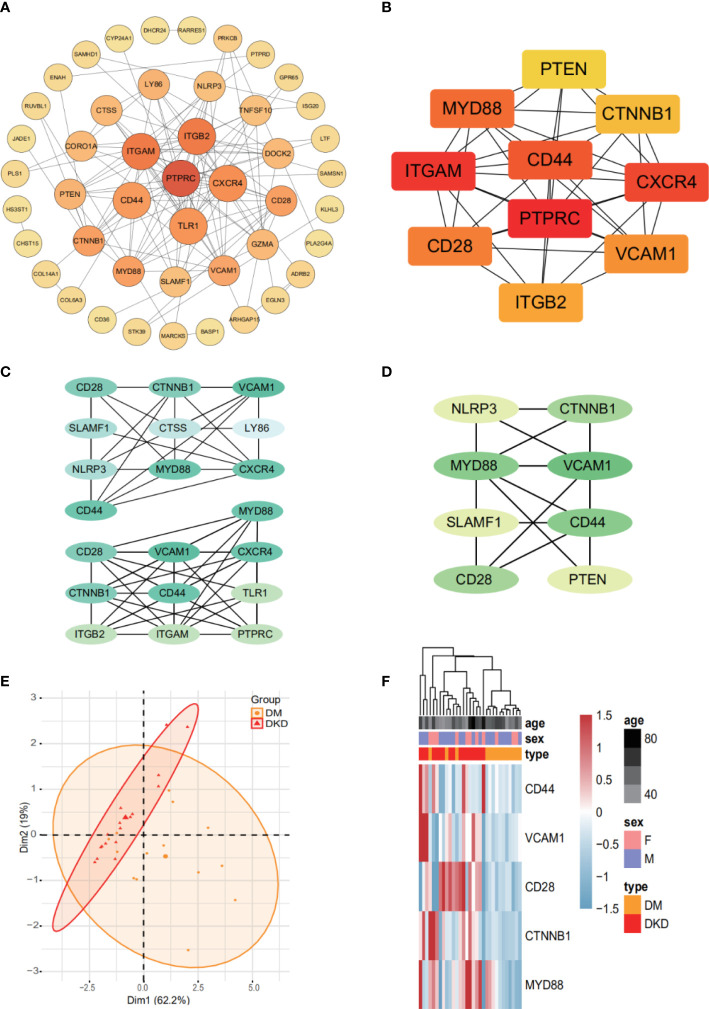
The final 5 genes panel could well distinguish DKD from DM. **(A)** Based on database STRING and Cytoscape software, PPI networks of 72 DhMGs were constructed. The darker the color of the node, the greater the degree value. **(B)** Hub genes (TOP10) selection and analysis performed by the MCC Algorithm. **(C)** Hub genes (TOP10) selection and analysis performed by the DMNC (top), and MNC (bottom) algorithms. **(D)** Module with an MCODE score of 4.8. **(E)** PCA plots showing DM (orange) and DKD (red) cfDNA cohorts using 5 genes panel as features. **(F)** Heatmaps of 5 genes panel with sample type, age, and sex information labeled in our cohort. Unsupervised hierarchical clustering was performed across genes and samples.

**Figure 5:**
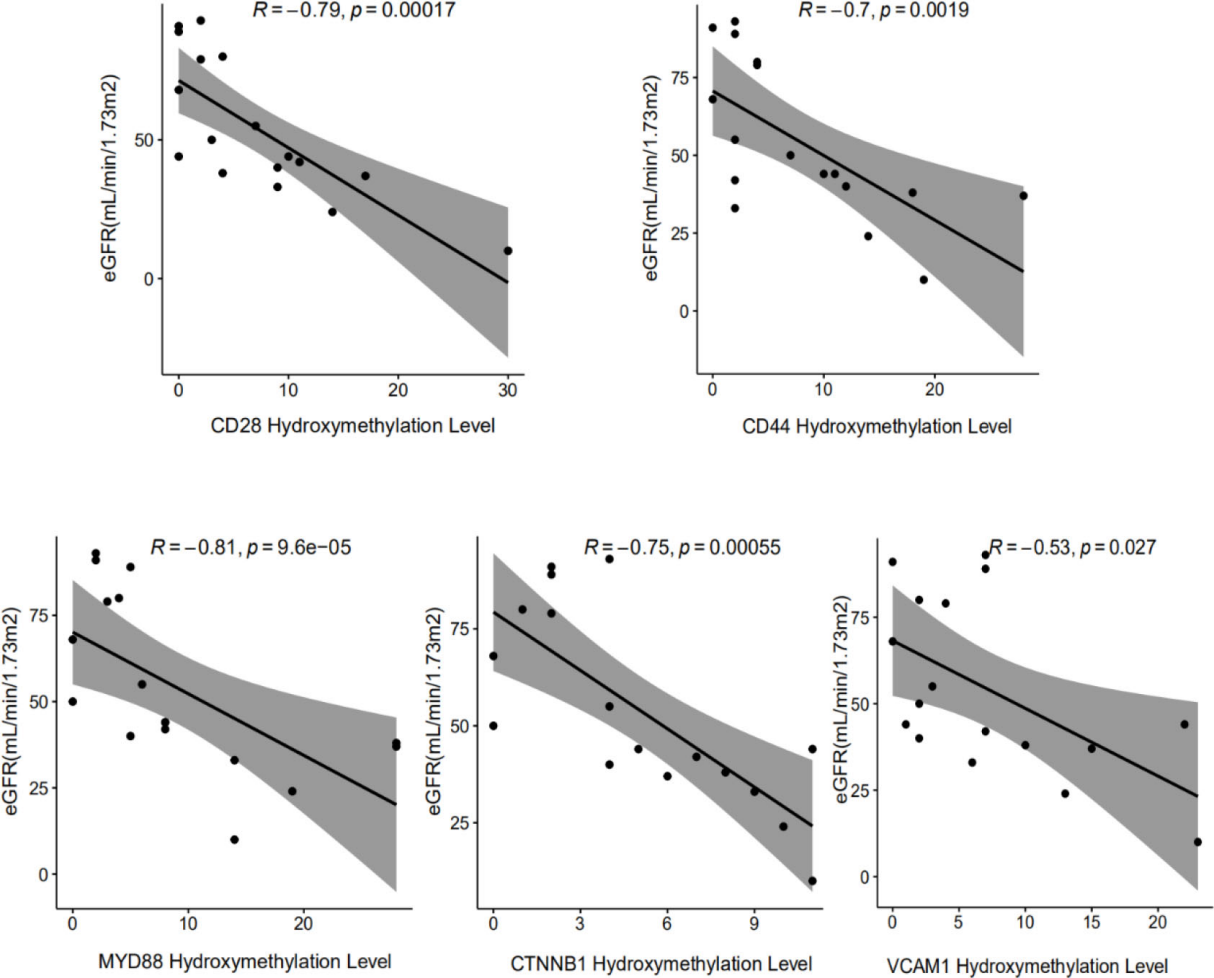
Correlation analysis between the hydroxymethylation level of cfDNA derived 5 DhMGs and the clinical parameters in DKD patients. The significant negative correlation could be found among the hydroxymethylation level of CD28, CD44, CTNNB1, MYD88, VCAM1 with eGFR.

Protein-protein interaction network analysis is a reliable method in understanding the biological responses in health and disease. Herein, protein interactions of the identified 72 markers were analyzed using the STRING database. A total of 46 nodes and 124 edges were revealed with combined scores>0.4 and visualized using Cytoscape software (**Figure 4A**). The Cytoscape plug-in cytoHubba including the MCC (**Figure 4B**), DMNC (**Figure 4C**, top), and MNC (**Figure 4C**, bottom) algorithms were applied to select hub genes, and the top 10 genes were selected by each of the three methods. The MCODE plug-in identified two desnsely connected modules, resulting in 8 markers with an MCODE score >4.8 as a screening criterion (**Figure 4D**, Additional file: **Supplementary Table 2**). The final 5 hub genes were obtained by intersecting the 8 markers identified in the densely connected modules with the top 10 hub genes discovered by the three methods. PCA analysis (**Figure 4E**) using the 5 genes panel separated DKD well from DM with a small overlap, which was also reasonable as DKD is developed from DM. Further, heatmap analysis showed that the final genes panel could well distinguish DKD from DM in our cohorts (**Figure 4F**). The boxplot of 5 hub genes were CD28 molecule (CD28), CD44 molecule (CD44), MYD88 innate immune signal transduction adaptor (MYD88), vascular cell adhesion molecule 1 (VCAM1) and CTNNB1, which were grouped by samples type (**Supplementary Figure S2**). The correlation analysis of 5 individual genes and clinical detection indicators, such as eGFR, creatinine, serum urea, UACR and 24-h urine protein, were performed. Data revealed that 5 hub genes were significantly negatively correlated with eGFR (**Figure 5**). Other indicators have no obvious correlation, and the results are not shown.

### Use of mice for verification

**Figure 6:**
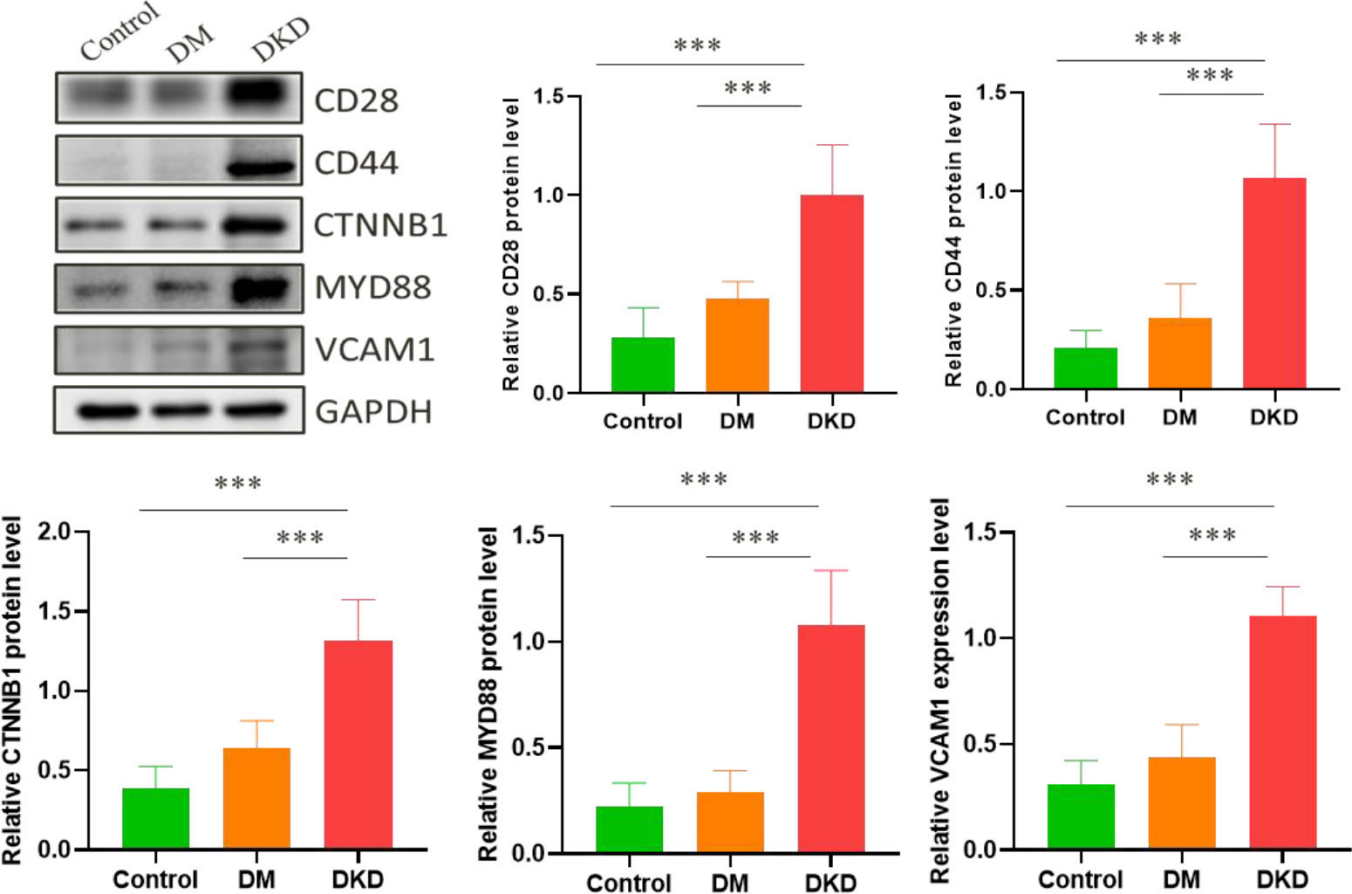
Related protein expression levels in mice kidney tissue. (***p < 0.001).

The renal injury index data of DKD mice model are shown in **Supplementary Table S1**. We detected the protein expression levels of CD28, CD44, CTNNB1, MYD88, and VCAM1 in the mice kidney tissue, and found that these 5 hub genes were all remarkably overexpressed in the kidney tissue of the DKD mice (**Figure 6**). The result suggests that the overexpression of 5 hub genes may play a critical role in DKD mechanism.

### Drug-gene interaction network

**Figure 7:**
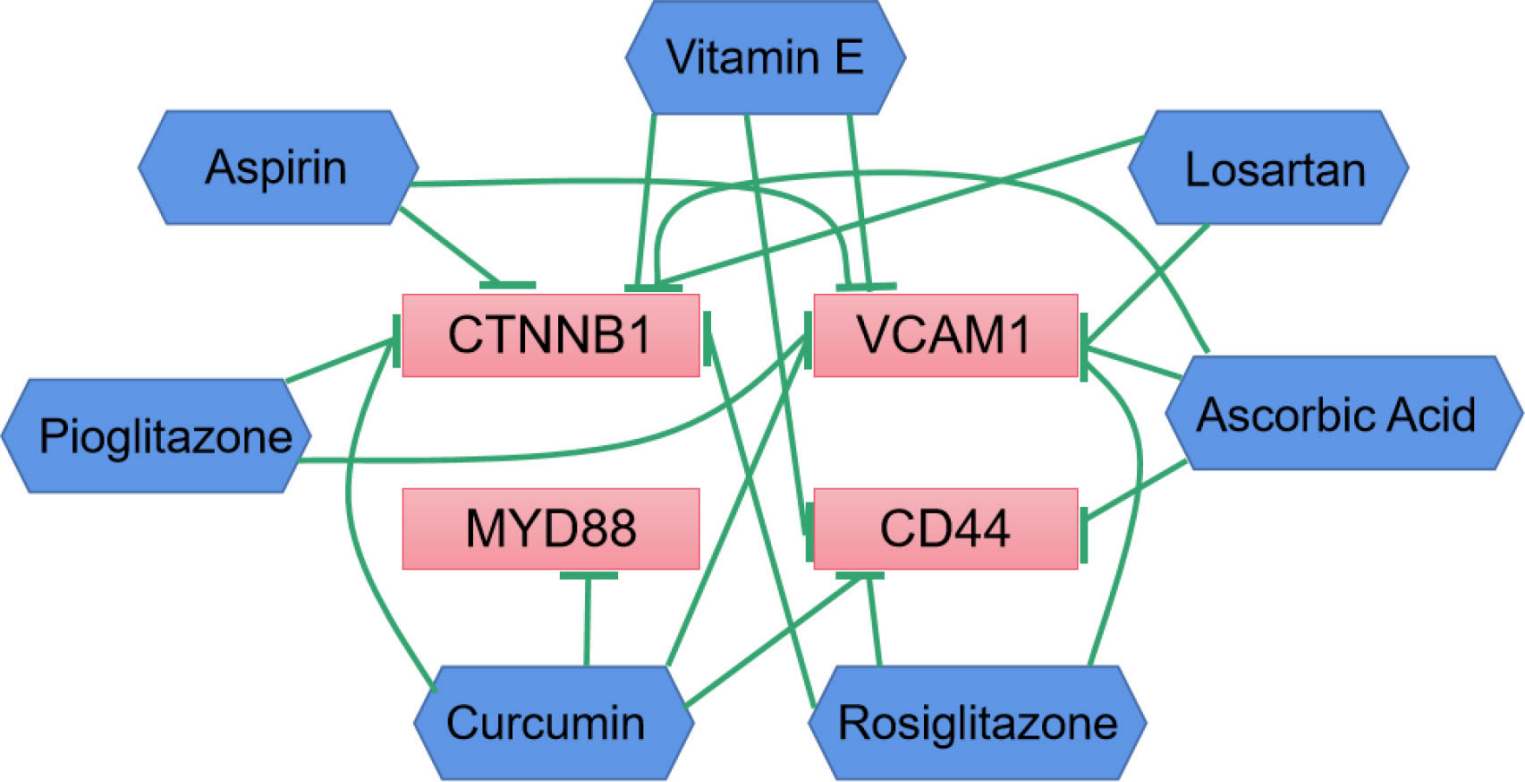
Drug-gene interactions network with drugs (blue) and 4 hub genes (red) was constructed using the CTD database. The green arrows represent that the drugs will decrease the expression of the hub genes.

To explore the interaction information between hub genes and DKD therapeutic drugs, we used CTD database to construct drug-gene interaction network. Various drugs for the treatment of DKD could affect the mRNA expression levels of CD44, CTNNB1, MYD88 and VCAM1 (**Figure 7**). Curcumin can significantly reduce the expression levels of these four genes. However, the relationship between drugs and CD28 has not been reported.

## Discussion

This study was focused on the discovery of hydroxymethylation-based biomarkers in plasma cfDNA and the possible involvement of epigenetics in the immunopathology of DKD. The availability of the blood samples and the convenience of cfDNA isolation makes it possible to monitor the occurrence and progression of the diseases with our technique. Thus, it may contribute to the personalized treatment and management of the disease.

Our data proved that the 5hmC signals were enriched in the promoter, exons, UTR, and TTS regions. Significant differences were found between DM and DKD for each genomic feature type, which suggested the crucial roles of these regions in the regulation of gene expression through 5hmC (16). At the same time, we found that these differences may be caused by the reduction of TET2 in patients with DKD (**Supplementary Figure S3**), which plays an important role in the conversion of 5-mC to 5-hmC. A number of DhMGs derived from plasma cfDNA was detected by differential analysis. Additionally, we observed markedly increased levels of IL-6 hydroxymethylation in plasma cfDNA from DKD patients, and IL-6 is thought to be a major stimulator of C-reactive protein (CRP) production in the liver. CRP is a very sensitive marker of inflammation (29). Notably, elevated serum CRP levels are closely associated with increased microalbuminuria and renal dysfunction in patients with DKD suggesting that CRP is closely related to the occurrence of diabetic kidney damage (30). Although IL-6 and CRP concentration were not measured in our collected clinical samples, but we believe that inflammation plays a role in the development of DKD. Furthermore, alignment with the transcriptional sequencing data from corresponding glomerular and tubular tissue further identified 72 overlapping markers. GO analysis of the 72 markers suggested the enrichment of immune cell differentiation and proliferation, endothelial cell differentiation, and toll-like receptor-related signaling pathways. Previous studies (31, 32) have shown that activation of innate immune cells and resident kidney cells can trigger kidney inflammation, thereby promoting the pathogenesis and progression of DKD. Toll-like receptors detect endogenous risk-related molecular patterns produced during diabetes and induce inflammatory responses in the sterile tubules through the NF-κB signaling pathway. Therefore, anti-inflammatory therapies may have renal protection for DKD. Interestingly, KEGG pathways demonstrate the enrichment of DhMGs for PD-L1 expression and PD-1 checkpoint pathway, Fc gamma R-mediated phagocytosis, Leukocyte transendothelial migration, which have been related with immunosuppression processes (33, 34).

By constructing a PPI network and further analyzing it, we identified key hub genes, including CTNNB1, VCAM1, MYD88, CD28, CD44. These 5 hub genes exhibited good clustering between DM and DKD. Notably, the hydroxymethylation levels of the 5 hub genes were identified to have a significant negative correlation with eGFR, which reflected the degree of renal injury. Additionally, we verified the protein expression levels of the 5 hub genes in the kidney tissues of C57BL/6 mice with renal injury. It was found that C57BL/6 mice were the mouse strains that were easy to replicate the human phenotype of DKD, so we used C57BL/6 mice for modeling (35). Western blot data revealed positive correlations of protein expression levels with the hydroxymethylation levels in plasma cfDNA and the transcriptome mRNA levels. It has been reported that 5hmC may be positively correlated with gene expression (36). In combination with RNA-seq, researchers have shown that the expression of genes with 5hmC on their gene bodies was significantly higher than the median of all genes, indicating that 5hmC on gene bodies may have profound effects on gene expression. Therefore, we speculated that the 5hmC modifications on gene bodies may be involved in the pathogenesis of DKD by regulating the gene expression. Even though more comprehensive evidences are still needed to fully support the correlation between 5hmC modification levels and protein expression levels, our present data in DKD, or at least the 5 identified hub genes, strongly suggested a positive correlation between 5hmC modification and protein expression.

To our knowledge, β-Catenin (CTNNB1) plays key role in cell adhesion and regulates Wnt-mediated transcription. In the nucleus, CTNNB1 binds to lymphocyte enhancer factor-1/T cell factor transcription factors to mediate gene transcription. In addition, genome-wide association studies indicated a correlation between transcription factor 7 like 2 (TCF7L2) polymorphism and the development of diabetes and DKD (37, 38). Previous research (39) revealed increased expression of Wnt/β-catenin pathway transcripts and proteins in glomeruli and podocytes of patients and mouse models of DKD. Up-regulation of the Wnt/β-catenin pathway in DKD may not only promote podocyte survival but also lead to cell detachment and podocyte loss. However, it’s also been reported that down-regulation of the Wnt/β-catenin pathway in podocytes might be important for terminal differentiation; however, it enhances apoptosis susceptibility. Therefore, we believe that balanced CTNNB1 expression is critical for glomerular filtration barrier maintenance in DKD. Meanwhile, in the correlation analysis, we found that the level of the epigenetic marker VCAM1 in the plasma cfDNA of DKD patients was highly negatively correlated with the level of eGFR, which was in consistent with the previous report (40)that renal filtration function gradually declines with the level of plasma VCAM1 increases. VCAM1 (Vascular Cell Adhesion Molecule 1) mainly encodes a cell surface sialoglycoprotein expressed by cytokine-activated endothelium and mediates leukocyte-endothelial cell adhesion and signal transduction that has been extensive-studied in cardiovascular diseases (41, 42). The high expression of VCAM1 in renal tubular cells may promote their interactions with immune cells to affect infiltration, thereby promoting fibrosis (43). At the same time, *in vitro* studies confirmed that the expression of VCAM1 was increased in HK-2 cells under high glucose conditions, and disulfiram could decrease the expression of VCAM1, the release of inflammatory cytokine, and fibrosis. It was reported that 1,25(OH)_2_D_3_ (44) inhibits inflammatory cell infiltration and tubulointerstitial fibrosis by downregulating the TLR4-MyD88-NF-κB pathway and thus exhibits protective effects on DKD. However, the drugs reported in these literatures are only preliminary studies. Then based on the results of the interaction between the currently clinical medication for the DKD and the hub genes, we found that various drugs could influence the expression levels of CD44, CTNNB1, VCAM1, MYD88. But CD28 has not been reported yet. However, studies have reported that DKD patients have increased infiltration of macrophages and T lymphocytes, so we reasonably suspected that CD28 acts as a T-cell-specific surface glycoprotein, which can interact with B7-1 (CD80) and B7-2 (CD86). It affects the proliferation, differentiation, and survival of T cells, thereby aggravating renal injury. It is worth noting that further experiments are needed to support whether DKD patients with hub gene overexpression can benefit from hub gene inhibition or whether these hub genes may become targets of drug treatment need ulteriorly biological experiments.

Yet, there are limitations to our study. Firstly, the number of DKD patients is relatively small. Larger scale, independent cohorts will be necessary to refine and validate the genes panel. Secondly, because mice kidney tissue samples were used to verify previous results of human, the results may be skewed by species origin. Finally, although previously related studies have explored part of the functions of these hub genes, it still lacks the epigenetic mechanisms of the regulation of DKD by these hub genes.

## Conclusions

In summary, the 5hmC-Seal assay was successfully applied in the cfDNA samples from a cohort of DM patients with or without DKD. Our study suggests that DhMGs may be used to uncover relevant mechanisms of inflammation and immune dysregulation in DKD. Altered 5hmC markers(CD44, CD28, CTNNB1, MYD88, VCAM1)were found in association with DKD, and that may contribute to gene regulation. Further data analysis indicated that cfDNA 5hmC signatures profiling might serve as a non-invasive epigenetic tool to distinguish DKD and to monitor DKD progression, provides new insight for the future molecularly targeted therapy of DKD.

## Data availability statement

The data presented in the study are deposited in online repositories. The names of the repository/repositories and accession number(s) can be found in the article/**Supplementary Material**.

## Ethics statement

The studies involving human participants were reviewed and approved by Ethics Committee of Peking University Third Hospital. The patients/participants provided their written informed consent to participate in this study. The animal study was reviewed and approved by Laboratory Animal Center, Peking University School of Medicine.

## Author contributions

J-LC, S-HB and YH conceived and designed the experiments. H-YC participated in library construct and 5hmC sequence with the help of R-YM and L-WD. J-LC analyzed data with help from H-YC, X-YW and M-ZZ. S-HB, YH, LZ, Z-QY, YM, GB and MD recruited patients, collected blood and organized clinical in formation. J-LC, R-YM and YH wrote the manuscript with input and comments from LC, H-YC and X-YW. All authors read and approved the final manuscript.

## Funding

National Natural Science Foundation of China–Xinjiang Joint Fund (U1403322) for Excellent Young Scholars and Xinjiang Uygur Autonomous Region University Scientific Research Project (XJEDU2021I017) to L-LL. National Natural Science Foundation of China (No. 81974506, 81673486 and 81373405) and the Beijing Natural Science Foundation (No. Z200019 and 7172119) to LT.

## Acknowledgments

We would like to acknowledge the essential contributions of all staffs and students who participated in this work.

## Conflict of interest

The authors declare that the research was conducted in the absence of any commercial or financial relationships that could be construed as a potential conflict of interest.

## Publisher’s note

All claims expressed in this article are solely those of the authors and do not necessarily represent those of their affiliated organizations, or those of the publisher, the editors and the reviewers. Any product that may be evaluated in this article, or claim that may be made by its manufacturer, is not guaranteed or endorsed by the publisher.

## Abbreviations

DM, diabetes mellitus; DKD, diabetic kidney disease; eGFR, estimated glomerular filtration rate; cfDNA, cell-free DNA; 5-hmC, 5-hydroxymethylcytosine; DEGs, differentially expressed genes; DhMGs, differentially hydroxymethylated genes; ADA, American Diabetes Association; UACR, urinary albumin-to-creatinine ratio; PPI, protein-protein interaction; PCA, principal component analysis; STRING, search tool for the retrieval of interacting genes; TSS, transcription start sites;

TTS, transcription termination sites; CRP, C-reactive protein; MCC, maximal clique centrality; MNC, maximum neighborhood component; DMNC, density of maximum neighborhood component.
