# Supplementary material for: 5-Hydroxymethylcytosine profiles in plasma cell-free DNA reflect molecular characteristics of diabetic kidney disease
Source: Front Endocrinol (Lausanne). 2022 Jul 29;13:910907. doi: 10.3389/fendo.2022.910907 (PMC9372268; doi:10.3389/fendo.2022.910907)
Supplement: Supplementary file 1 [file DataSheet_1.docx]

**Supporting information**

5-Hydroxymethylcytosine profiles in plasma cell-free DNA reflect molecular characteristics of diabetic kidney disease

Jin-Lin Chu†^1,2^, Shu-Hong Bi†^3^, Yao He†^4^, Rui-Yao Ma^1,2^, Xing-Yu Wan^5,6^, Zi-Hao Wang^8^, Lei Zhang^5,6^, Meng-Zhu Zheng^5,6^, Zhan-Qun Yang^5,6^, Ling-Wei Du^7^, Yiminiguli Maimaiti^1,2^, Gulinazi Biekedawulaiti^1,2^, Maimaitiyasen Duolikun^1,2^, Hang-yu Chen^5,6^, Long Chen^5,6^, Lin-Lin Li*^1,2^, Lu Tie*^4^, Jian Lin*^5,6^


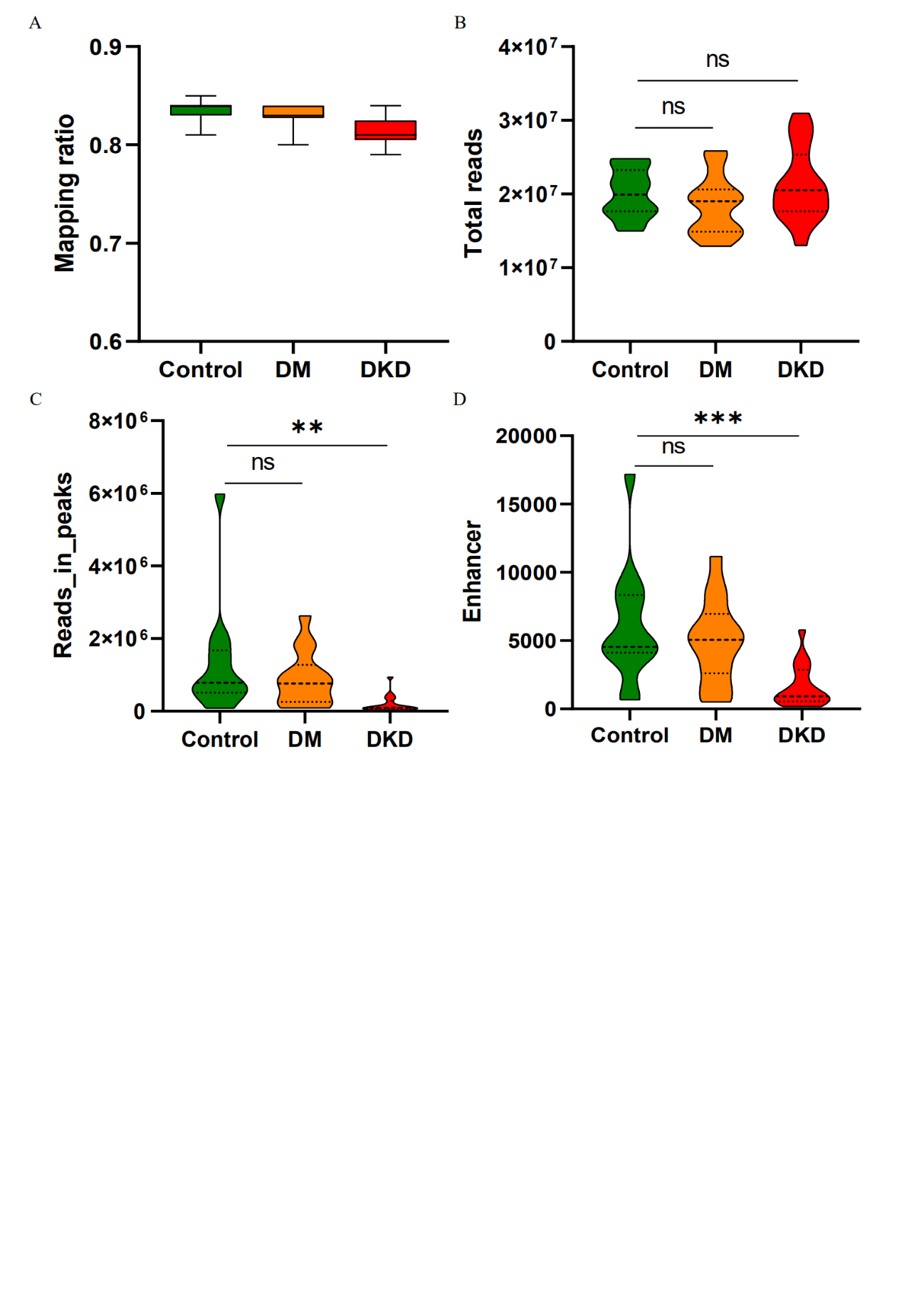


**Fig S1.** Three sets of samples (Control, DM, DKD) sequencing data quality control (QC) plots such as mapping rate, total reads, reads in peaks and peaks in enhancer. ***p*<0.01, ****p*<0.001


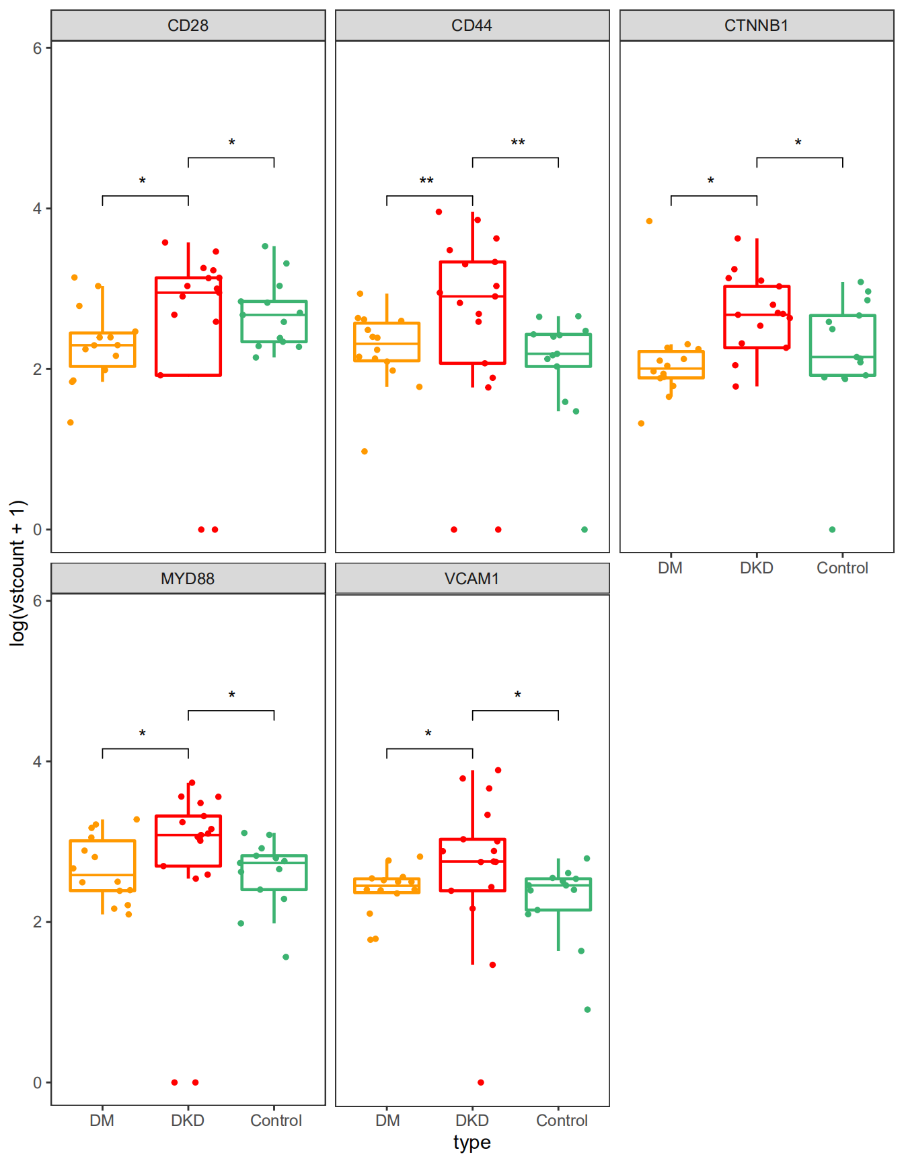


**Fig S2.** Boxplots of 5 genes panel in our cohort (Control vs. DM vs. DKD). Log2 transformed of TMM normalized 5hmC enrichment values were plotted, and the Wilcoxon t-test was used. **p*<0.05, ***p*<0.01

**Table S1. Basic detection indicators of model C57BL/6 mice** (‾x±SD )

|  | Control | 8week | 16week |
| --- | --- | --- | --- |
| Weight (g) | 30.44±1.25 | 27.95±1.21^**^ | 27.92±1.64^**^ |
| Glucose (mmol/L) | 6.62±0.60 | 30.03±4.28^**^ | 32.75±0.93^**^ |
| Urine protein (mg/g) | 3.08±1.66 | 49.54±21.69^**^ | 1248.25±826.98^*#^ |
| Serum creatinine (μM) | 20.02±1.20 | 20.95±3.09 | 62.96±12.68^**##^ |
| Urea nitrogen (mmol/L) | 11.77±1.85 | 13.74±1.14 | 22.18±4.08^**##^ |

Note: Compared with Control, **p*＜0.05, ***p*＜0.01; Compared with 8 week, ^#^*p*＜0.05, ^##^*p*＜0.01


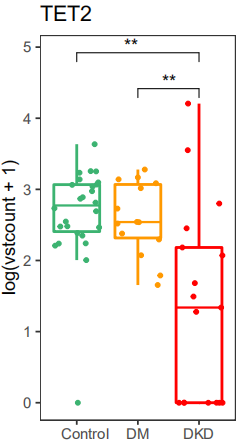


**Fig S3.** Boxplots of TET2 in our cohort (Control vs. DM vs. DKD). Log2 transformed of TMM normalized 5hmC enrichment values were plotted, and the Wilcoxon t-test was used. ***p*<0.01
